# Predictors of Critical Care Interventions and Length of Stay in Pediatric Sickle Cell Acute Chest Syndrome: A Five-Year Pediatric Intensive Care Unit (PICU) Cohort Study

**DOI:** 10.7759/cureus.104293

**Published:** 2026-02-26

**Authors:** Aseel Albalawi, Ehab Hanafy, Mustafa M Altoonisi, Mohammed Mustafa, Wessam Soliman, Mohammed Kamal, Ahmed M Karkar, Mohammed Elhady

**Affiliations:** 1 Pediatric Department, King Salman Armed Forces Hospital, Tabuk, SAU; 2 Oncology Center, Armed Forces Hospital Southern Region, Khamis Mushayt, SAU; 3 Prince Sultan Oncology Center, King Salman Armed Forces Hospital, Tabuk, SAU

**Keywords:** acute chest syndrome, exchange transfusion, length of stay, mechanical ventilation, pediatric intensive care, saudi arabia, sickle cell anemia

## Abstract

Introduction

Acute chest syndrome (ACS) remains one of the most severe complications of pediatric sickle cell anemia (SCA) and a leading cause of Pediatric Intensive Care Unit (PICU) admission. Despite its clinical significance, regional data describing PICU-level characteristics, interventions, and outcomes in children with SCA are limited. This study aimed to evaluate the clinical profile, management strategies, and outcomes of pediatric SCA patients admitted to the PICU at a tertiary care center in Tabuk, Saudi Arabia.

Methods

We conducted a retrospective cohort study of pediatric patients aged one to 14 years with confirmed SCA admitted to the PICU for ACS between January 2020 and January 2025 (five-year period) at King Salman Armed Forces Hospital, Tabuk. Demographic data, laboratory parameters, prior disease history, PICU interventions, and clinical outcomes were extracted from medical records. Associations between clinical predictors and major interventions were evaluated using chi-square/Fisher’s exact testing. Predictors of PICU length of stay were analyzed using comparative statistical methods.

Results

Forty-one patients were included (mean age 8.71 ± 3.54 years); 25 (61.0%) were male. Most patients had the homozygous sickle cell disease (HbSS) genotype (36 (87.8%)), and 30 (73.2%) were receiving hydroxyurea. Exchange transfusion was performed in 29 (70.7%) admissions, while mechanical ventilation was required in two (4.9%). Exchange transfusion was not significantly associated with demographic or baseline clinical variables. Although Fisher’s exact testing demonstrated statistical significance for prior ICU admission (p = 0.034) and prior stroke (p = 0.007), the very small number of ventilation events (n = 2) limits the robustness of these associations. These findings should therefore be interpreted with caution. Mean PICU length of stay was 3.24 ± 2.80 days and was significantly longer among patients with a history of stroke (p = 0.003). In-PICU complications were uncommon.

Conclusion

In this five-year single-center retrospective cohort of pediatric patients with SCA admitted to the PICU for ACS, exchange transfusion was frequently utilized, whereas invasive mechanical ventilation was uncommon. A signal toward greater PICU severity was observed among patients with prior neurologic events or prior ICU exposure; however, given the small sample size and limited number of ventilation events, these observations should be interpreted with caution. Larger, multicenter prospective studies are needed to further clarify risk stratification and inform evidence-based critical care planning in this population.

## Introduction

Sickle cell anemia (SCA) is a severe inherited hemoglobinopathy caused by a β-globin gene mutation leading to hemoglobin S (HbS) polymerization, erythrocyte sickling, chronic hemolysis, and recurrent vaso-occlusion [1]. Despite advances in supportive care, SCA remains associated with significant morbidity and early mortality, particularly in childhood [2].

Acute chest syndrome (ACS) is one of the most serious and potentially life-threatening complications of SCA. It is defined by the presence of a new pulmonary infiltrate on chest imaging accompanied by fever and/or respiratory symptoms [3]. ACS represents the second most common cause of hospitalization in children with SCA and remains a leading cause of disease-related mortality [3,4]. Recurrent ACS episodes are associated with long-term pulmonary dysfunction and increased mortality risk [5].

The pathophysiology of ACS is multifactorial and includes infection, fat embolization from bone marrow necrosis, pulmonary infarction, hypoventilation due to pain, and inflammatory cascades that exacerbate vaso-occlusion within the pulmonary microvasculature [3,6]. Identified risk factors for severe ACS include elevated leukocyte counts, prior ACS episodes, asthma, and lower baseline hemoglobin levels [3,7].

Management of ACS is largely supportive and includes oxygen therapy, incentive spirometry, antibiotics, simple or exchange transfusion, and ventilatory support when necessary [3,8]. Exchange transfusion plays a central role in severe ACS by reducing HbS concentration and improving oxygen delivery [9]. However, high-quality randomized data guiding critical care management remain limited [10].

Although several international studies have described ACS epidemiology and management patterns [3,11], detailed PICU-level analyses - particularly within Middle Eastern populations - remain scarce. Saudi Arabia has a significant burden of SCA, with regional variation in prevalence and clinical phenotype [12]. A recent tertiary-center study in Saudi Arabia highlighted the burden of pediatric ACS but did not focus specifically on PICU-level interventions and predictors of critical care outcomes [13].

Understanding determinants of intensive care interventions and length of stay in pediatric ACS is essential for optimizing risk stratification, early escalation of care, and resource allocation. Therefore, this five-year retrospective cohort study (2020-2025) evaluates clinical characteristics, critical care interventions, and outcomes of pediatric SCA patients admitted with ACS to a tertiary PICU in Tabuk, Saudi Arabia.

## Materials and methods

Study design and setting

This study was designed as a retrospective cohort study conducted at King Salman Armed Forces Hospital, Tabuk, Saudi Arabia. The hospital is a tertiary care center providing specialized pediatric hematology and critical care services. The study evaluated pediatric patients with SCA admitted to the Pediatric Intensive Care Unit (PICU) with ACS over a five-year period from January 1, 2020, to January 1, 2025.

Study population and sampling

The study population included all pediatric patients aged one to 14 years with a confirmed diagnosis of SCA who were admitted to the PICU with ACS during the study period. A total population sampling technique was employed, whereby all eligible cases meeting the inclusion criteria within the defined timeframe were included. No sampling reduction or randomization was applied.

ACS was defined according to established criteria as a new pulmonary infiltrate on chest imaging accompanied by fever and/or respiratory symptoms. This study did not apply a formal ACS severity grading system (e.g., oxygen requirement stratification or radiologic scoring). All pediatric patients admitted to the PICU with a primary diagnosis of ACS during the study period were included, regardless of clinical severity classification. PICU admission was based on treating physician judgment and institutional escalation criteria rather than predefined severity scoring.

Inclusion criteria

Eligible participants were pediatric patients aged one to 14 years at the time of admission with a confirmed diagnosis of SCA (homozygous sickle cell disease (HbSS) or sickle β-thalassemia) who were admitted to the PICU at King Salman Armed Forces Hospital, Tabuk, between January 2020 and January 2025 with a primary diagnosis of ACS.

Exclusion criteria

Patients were excluded if they did not have a confirmed diagnosis of SCA, if their PICU admission was unrelated to ACS, or if their medical records were incomplete or lacked essential clinical, laboratory, or outcome data required for analysis.

Data collection instrument

Data were collected retrospectively from electronic and paper-based medical records, including the hospital information system and laboratory databases. A structured data extraction framework was utilized to ensure standardized and consistent retrieval of information. Variables collected included demographic characteristics (age and sex), prior clinical history (previous ICU admission, previous ACS, previous stroke, frequency of vaso-occlusive crisis admissions, hydroxyurea therapy, and regular transfusion program), laboratory parameters at admission (hemoglobin, mean corpuscular volume, white blood cell count, platelet count, C-reactive protein (CRP), fetal hemoglobin (HbF) percentage, and HbS percentage), and PICU-related variables (source of admission, indication for admission, invasive procedures, transfusion modality, respiratory support, in-PICU complications, and length of PICU stay). All data were anonymized prior to statistical analysis to maintain confidentiality.

Data analysis

Statistical analysis was performed using IBM Statistical Package for the Social Sciences (SPSS), version 26.0 (IBM Corp., Armonk, NY). Continuous variables were presented as mean ± standard deviation, with minimum and maximum values reported where applicable. Categorical variables were expressed as frequencies and percentages. Associations between categorical variables and major interventions (exchange transfusion and mechanical ventilation) were evaluated using chi-square tests or Fisher’s exact tests when appropriate. Comparisons of mean PICU length of stay across predictor variables were conducted using independent sample t-tests or one-way analysis of variance (ANOVA), as appropriate. A two-tailed p-value of less than 0.05 was considered statistically significant.

Ethical considerations

Ethical approval was obtained from King Salman Armed Forces Hospital Research and Ethics Committee. The study followed the principles of the Declaration of Helsinki (2013 revision) and local institutional research governance guidelines.

A waiver of informed consent was granted, as the study involved no collection or disclosure of personally identifiable patient information and posed no risk to participant privacy or confidentiality.

## Results

A total of 41 pediatric patients with SCA were admitted to the PICU during the study period. Baseline characteristics are summarized in Table 1. The mean age was 8.71 ± 3.54 years (range, one to 14 years), with nearly half of the cohort aged 10-14 years (19, 46.3%). Patients aged five to nine years accounted for 16 (39.0%), and younger children aged one to four years represented six (14.6%). Males predominated, comprising 25 (61.0%) of the study population.

**Table 1:** Baseline Demographic, Laboratory, and Clinical Characteristics of Pediatric Patients With Sickle Cell Anemia Admitted to the PICU (N = 41) Values are presented as number (N), minimum, maximum, mean, and standard deviation (SD). C-reactive protein (CRP) values were available for 33 patients. Hemoglobin (HB) is expressed in g/dL; mean corpuscular volume (MCV) in femtoliters (fL); white blood cell count (WBC) in ×10⁹/L; platelet count (PLT) in ×10⁹/L; CRP in mg/L; fetal hemoglobin (HbF) and hemoglobin S (HbS) as percentages (%). Length of stay represents the total duration of Pediatric Intensive Care Unit admission in days.

	Normal Range	N	Minimum	Maximum	Mean	Standard Deviation
Age	Children, 1-14 years	41	1	14	8.71	3.537
HB	11.5-15.5 g/dL	41	5.3	10.8	7.6683	1.24126
MCV	75-90 fL	41	59.2	97.9	77.7732	8.4882
WBC	4.5-13.5 × 10⁹/L	41	3.6	63.6	21.1949	12.26643
PLT	150-450 × 10⁹/L	41	38	665	326.4878	182.561
CRP	< 5 mg/L	33	0.12	35.6	13.8339	8.93713
HBF	< 2%	41	2	39	11.2829	7.71404
HBS	0	41	17	92.2	69.82195	16.64453
Length of stay (days)	-	41	1	14	3.2439	2.79983

At admission, laboratory findings reflected the expected hematologic profile of sickle cell disease. The mean hemoglobin level was 7.67 ± 1.24 g/dL, and the mean white blood cell count was elevated at 21.19 ± 12.27 ×10⁹/L. CRP was available for 33 (80.5%) patients and demonstrated an inflammatory pattern, with a mean value of 13.83 ± 8.94 mg/L. Hemoglobin electrophoresis showed a mean HbF of 11.28 ± 7.71% and HbS of 69.82 ± 16.64%. The overall mean PICU length of stay was 3.24 ± 2.80 days.

Clinical features are presented in Table 2. Most patients had the HbSS genotype (36, 87.8%). A prior ICU admission was documented in eight (19.5%), while 12 (29.3%) had a history of previous ACS. A history of stroke was present in four (9.8%), and recurrent vaso-occlusive crises (≥2 admissions) were common, occurring in 34 (82.9%) patients. Hydroxyurea therapy was being used by 30 (73.2%), whereas only three (7.3%) were on a regular transfusion program.

**Table 2:** Clinical Characteristics, Interventions, and Outcomes of Pediatric Sickle Cell Anemia Patients Admitted to the PICU (N = 41) Data are presented as n (%). Percentages are calculated using the total study population (N = 41) as the denominator. All patients who received invasive mechanical ventilation had previously received non-invasive ventilatory support. ACS, acute chest syndrome; CVP, central venous catheter; DVT, deep vein thrombosis; ER, emergency room; HbSS, homozygous sickle cell disease; ICU, intensive care unit; SC β-thal, sickle cell beta-thalassemia; VOC, vaso-occlusive crisis

		Frequency	Percentage
Age group	1-4 years	6	14.6
	5-9 years	16	39
	10-14 years	19	46.3
Gender	Male	25	61
	Female	16	39
Previous ICU	No	33	80.5
	Yes	8	19.5
Previous ACS	No	29	70.7
	Yes	12	29.3
Previous stroke	No	37	90.2
	Yes	4	9.8
2+ admissions VOC	No	7	17.1
	Yes	34	82.9
Hydroxyurea	No	11	26.8
	Yes	30	73.2
Regular transfusion	No	38	92.7
	Yes	3	7.3
Primary diagnosis and concurrent conditions at PICU admission	ACS	35	85.4
	ACS, girdle syndrome	1	2.4
	ACS, hepatic sequestration	1	2.4
	ACS, VOC	3	7.3
	Stroke	1	2.4
Complications at PICU	Osteomyelitis	1	25
	ACS, DVT	1	25
	Headache	1	25
	Splenic sequestration	1	25
Procedure	None	4	9.8
	CVP	36	87.8
	Chest tube, CVP	1	2.4
Exchange transfusion	No	12	29.3
	Yes	29	70.7
Simple transfusion	No	26	63.4
	Yes	15	36.6
Diagnosis	HbSS	36	87.8
	SC β-thal	5	12.2
Admission source	Er	8	19.5
	Clinic	1	2.4
	Station	32	78
Assisted ventilation	No	39	95.1
	Yes	2	4.9
Ventilation	No	39	95.1
	Yes	2	4.9

ACS alone was the principal indication for PICU admission in 35 (85.4%) cases, with smaller proportions presenting with combined ACS and other complications. Most admissions originated from inpatient wards (32, 78.0%), reflecting clinical deterioration during hospitalization rather than initial emergency presentation.

In terms of PICU management, central venous catheter insertion was performed in 36 (87.8%) patients. Exchange transfusion was a central therapeutic intervention, administered to 29 (70.7%) patients, whereas simple transfusion was given to 15 (36.6%). Two patients (4.9%) required non-invasive ventilatory support and subsequently progressed to invasive mechanical ventilation.

Complications during PICU stay were rare and occurred in isolated cases, including osteomyelitis, deep vein thrombosis associated with ACS, headache, and splenic sequestration.

Analysis of associations between clinical characteristics and interventions is detailed in Table 3. Exchange transfusion was broadly utilized across demographic and clinical subgroups, with no clear predictors identified. In contrast, the need for invasive ventilation was associated with prior ICU admission and prior stroke, suggesting that children with previous severe disease manifestations were more likely to experience respiratory decompensation.

**Table 3:** Association Between Clinical Variables and Major PICU Interventions Data are presented as n. A p-value < 0.05 was considered statistically significant. ACS, acute chest syndrome; Fisher, Fisher’s exact test; HbSS, homozygous sickle cell disease; ICU, intensive care unit; SC β-thal, sickle cell beta-thalassemia; VOC, vaso-occlusive crisis; χ², Pearson chi-square test

Outcome Variable	Predictor Variable	Category	No	Yes	Test Used	χ² value	p-value
Exchange	Age group	1-4 years	2	4	χ²	0.16	0.924
		5-9 years	5	11			
		10-14 years	5	14			
Assisted ventilation	Age group	1-4 years	6	0	Fisher	-	0.828
		5-9 years	15	1			
		10-14 years	18	1			
Ventilation	Age group	1-4 years	6	0	Fisher	-	0.828
		5-9 years	15	1			
		10-14 years	18	1			
Exchange	Gender	Male	6	19	χ²	0.86	0.354
		Female	6	10			
Assisted ventilation	Gender	Male	25	0	Fisher	-	0.146
		Female	14	2			
Ventilation	Gender	Male	23	2	Fisher	-	0.512
		Female	16	0			
Exchange	Previous ICU	No	9	24	χ²	0.33	0.568
		Yes	3	5			
Assisted ventilation	Previous ICU	No	31	2	Fisher	-	0.475
		Yes	8	0			
Ventilation	Previous ICU	No	33	0	Fisher	-	0.034
		Yes	6	2			
Exchange	Previous ACS	No	8	21	χ²	0.14	0.713
		Yes	4	8			
Assisted ventilation	Previous ACS	No	28	1	Fisher	-	0.509
		Yes	11	1			
Ventilation	Previous ACS	No	27	2	Fisher	-	0.351
		Yes	12	0			
Exchange	Previous stroke	No	11	26	Fisher	-	0.843
		Yes	1	3			
Assisted ventilation	Previous stroke	No	35	2	Fisher	-	0.634
		Yes	4	0			
Ventilation	Previous stroke	No	37	0	Fisher	-	0.007
		Yes	2	2			
Exchange	≥2 admissions VOC	No	3	4	χ²	0.75	0.386
		Yes	9	25			
Assisted ventilation	≥2 admissions VOC	No	7	0	Fisher	-	0.511
		Yes	32	2			
Ventilation	≥2 admissions VOC	No	7	0	Fisher	-	0.511
		Yes	32	2			
Exchange	Hydroxyurea	No	4	7	χ²	0.37	0.545
		Yes	8	22			
Assisted ventilation	Hydroxyurea	No	11	0	Fisher	-	0.380
		Yes	28	2			
Ventilation	Hydroxyurea	No	9	2	Fisher	-	0.067
		Yes	30	0			
Exchange	Diagnosis	HbSS	12	24	Fisher	-	0.298
		SC β-thal	0	5			
Assisted ventilation	Diagnosis	HbSS	34	2	Fisher	-	0.589
		SC β-thal	5	0			
Ventilation	Diagnosis	HbSS	34	2	Fisher	-	0.589
		SC β-thal	5	0			

Length of stay analysis is summarized in Table 4. PICU stay did not significantly differ across most demographic or clinical variables. However, patients with a history of stroke 4 (9.8%) had a markedly longer PICU stay (7.00 ± 4.83 days) compared with those without prior stroke 37 (90.2%) (2.84 ± 2.24 days). A trend toward shorter stay was observed among patients with prior ACS, although this did not reach statistical significance. No PICU or in-hospital deaths occurred during the study period.

**Table 4:** Predictors of PICU Length of Stay Among Pediatric Patients With Sickle Cell Disease Length of stay is presented as mean ± standard deviation (SD). A p-value < 0.05 was considered statistically significant. *Independent samples t-test. ^#^One-way analysis of variance (ANOVA). ACS, acute chest syndrome; HbSS, homozygous sickle cell disease; ICU, intensive care unit; SC β-thal, sickle cell beta-thalassemia; VOC, vaso-occlusive crisis

Variable		Average Length of Stay		Test Statistic	p-value
		Mean	SD		
Age group	1-4 years	2.17	2.86	0.513^#^	0.603
	5-9 years	3.38	3.24		
	10-14 years	3.47	2.44		
Gender	Male	2.92	3.01	0.924*	0.361
	Female	3.75	2.44		
Previous ICU	No	3.06	2.34	0.848*	0.401
	Yes	4.00	4.34		
Previous ACS	No	3.72	2.95	1.751*	0.088
	Yes	2.08	2.07		
Previous stroke	No	2.84	2.24	3.117*	0.003
	Yes	7.00	4.83		
2+ admissions VOC	No	2.86	2.48	0.397*	0.693
	Yes	3.32	2.89		
Hydroxyurea	No	3.91	4.04	0.919*	0.364
	Yes	3.00	2.23		
Diagnosis	HBSS	3.25	2.87	0.037*	0.971
	SC β-thal	3.20	2.49		

Overall, the results indicate that while exchange transfusion was frequently required, invasive ventilation was uncommon and primarily observed among patients with prior neurologic or critical care history.

## Discussion

This five-year single-center cohort provides important insight into the clinical course and critical care resource utilization of pediatric SCA patients admitted with ACS. Several key observations emerge from our findings.

First, exchange transfusion was performed in the majority of admissions. This reflects contemporary practice patterns for moderate-to-severe ACS requiring intensive care support. Exchange transfusion is known to reduce HbS concentration, improve oxygenation, and decrease blood viscosity, thereby mitigating ongoing pulmonary vaso-occlusion [9]. While randomized controlled trials specifically in PICU populations are lacking, observational data and guideline recommendations support early transfusion in severe cases [3,8]. The absence of significant demographic predictors of exchange transfusion in our cohort suggests that clinical severity rather than baseline patient characteristics likely drives transfusion decisions.

Second, invasive mechanical ventilation was required in a small proportion of cases. Historically, ACS has been associated with substantial respiratory failure risk, with reported mechanical ventilation rates ranging between 10 and 20% in some pediatric cohorts [3,11]. Our lower ventilation rate may reflect early intervention, aggressive transfusion practices, or institutional thresholds for escalation. Importantly, prior ICU admission and prior stroke were significantly associated with ventilation requirement. This finding is clinically meaningful. Prior ICU exposure may represent a marker of severe disease phenotype, while prior stroke reflects advanced vasculopathy and systemic endothelial dysfunction, both of which may predispose to pulmonary decompensation during ACS episodes [14,15].

Stroke in SCA is a marker of severe vasculopathy and systemic endothelial injury [14]. Patients with prior cerebrovascular events may exhibit impaired physiologic reserve, altered pulmonary vascular responsiveness, and higher inflammatory burden, potentially explaining both increased ventilation requirement and prolonged PICU stay observed in our cohort. The STOP trial and subsequent long-term studies emphasize that cerebrovascular disease in SCA reflects a high-risk phenotype with systemic implications beyond the central nervous system [15].

Third, the length of PICU stay was relatively short overall but significantly prolonged in patients with prior stroke. Length of stay is an important surrogate for disease severity and healthcare utilization. Prior literature has shown that ACS severity correlates with increased inflammatory markers and pulmonary involvement [3,7]. Although we did not perform multivariable regression due to sample size limitations, our findings suggest that neurologically high-risk children represent a subgroup requiring intensified monitoring and early escalation strategies.

Hydroxyurea therapy was common in our population. Hydroxyurea increases HbF levels and reduces the frequency of VOC and ACS episodes [16]. While hydroxyurea did not significantly influence acute PICU outcomes in our cohort, its high utilization likely reflects adherence to established guidelines and may partially explain relatively low ventilation rates. Long-term data demonstrate that hydroxyurea reduces hospitalization and ACS incidence, although its impact once ACS has already progressed to PICU admission remains less clearly defined [16].

The multifactorial etiology of ACS continues to complicate management. Infectious triggers remain common, and empirical antibiotic therapy is widely recommended despite limited high-quality randomized evidence defining optimal regimens [10]. The inflammatory nature of ACS suggests that systemic inflammatory burden may influence severity and progression [6,7]. Future studies incorporating biomarkers, such as CRP, lactate dehydrogenase (LDH), and inflammatory cytokine panels, may further refine risk stratification.

When contextualized within global data, our findings are consistent with prior large-scale ACS analyses, demonstrating high transfusion rates and variable ventilation needs [3,11]. Importantly, our study adds region-specific data from Saudi Arabia, contributing to the limited body of Middle Eastern PICU-focused SCA research. Regional genetic modifiers, healthcare infrastructure, and early intervention strategies may influence clinical trajectories.

Limitations

This study has limitations. Its retrospective design introduces potential information bias and limits causal inference. The single-center setting may limit generalizability. The relatively small sample size restricted multivariable modeling. Nevertheless, the strength of this study lies in the complete capture of a five-year PICU cohort with detailed intervention analysis.

Future multicenter studies should incorporate standardized severity scoring, multivariable risk modeling, and long-term pulmonary outcome follow-up. Prospective data collection would enable more precise evaluation of predictors of respiratory failure and prolonged critical care utilization.

## Conclusions

In this five-year retrospective cohort of pediatric patients with SCA admitted to the PICU for ACS, exchange transfusion was frequently utilized, whereas invasive mechanical ventilation was required in a minority of cases. Prior ICU admission and a history of stroke were significantly associated with increased likelihood of ventilatory support, and prior stroke was independently associated with prolonged PICU length of stay. These findings suggest that children with neurologic comorbidity or prior critical care exposure represent a higher-risk subgroup during ACS episodes.

Overall, ACS remains a major driver of intensive care utilization in pediatric sickle cell disease. Early identification of high-risk patients and timely implementation of targeted interventions may help optimize outcomes and resource allocation. Further multicenter, prospective studies are warranted to refine risk stratification models and establish evidence-based critical care protocols for this vulnerable population.
